# Editorial: Therapeutic modulators inhibiting neuromuscular and motor neuron degeneration

**DOI:** 10.3389/fnins.2023.1188945

**Published:** 2023-04-06

**Authors:** Sidharth Mehan

**Affiliations:** Division of Neuroscience, Department of Pharmacology, ISF College of Pharmacy, Moga, Punjab, India

**Keywords:** neurodegeneration, motor neuron disease, biomarkers, phytopharmaceuticals, neuroprotection

## Introduction

In recent decades, the global prevalence of brain illnesses has increased, as has the disease's socioeconomic impact. A rise in 5% of motor neuron disorders (MNDs) and neuromuscular degeneration is attributable to unidentified treatment, and no specific diagnostic biomarkers can prove the progression and prevention of these neuronal consequences (Alam et al., 2021; Kapoor and Mehan, 2021). MND is a chronic motor neuron disease that affects both the central and peripheral nervous systems, causing motor neuron degeneration as well as a variety of neurological impairments. Demyelination can occur in white matter in this condition, most notably in the Brain and spinal cord. Peripheral motor neurons are sometimes damaged, causing patients to be unable to execute motor functions in their daily lives (Kumar et al., 2021, 2023).

The number of patients with MND can double globally in forthcoming decades because of its idiopathic nature. Neurologists are striving to prevent and identify this motor impairment using a variety of developing therapy. MND is a major rising concern worldwide, and the number of patients is expanding by the day; this poses a significant challenge for physicians and pharmaceutical research organizations in treating, diagnosing, and confirming the severity of disease progression. It is estimated that 48.3% of females aged 20 to 50 years will develop MND in the next decades worldwide, where this disease is more vulnerable in females than males. To overcome these problems, novel molecular diagnosis biomarkers and potential preventive medicines for patients are required.

Although early and adequate MND therapy and diagnosis are difficult, no unique biomarker can confirm and aid in treatment and diagnosis. Some biomarkers are currently present in the cerebral spinal fluid (oligoclonal bands and immunoglobins such as IgG) and blood serum of MND patients; nevertheless, they are not conclusive proof of the disease. These diagnostic criteria also aid in identifying clinically comparable motor neuron and neurodegenerative illnesses. As a result, the most significant challenge in today's MND treatment spectrum is the lack of accurate biomarkers to identify the appropriate medicines for motor neuron patients. As a result, researchers are establishing the role of many molecular markers in the diagnosis and their potential modulators to prevent neuronal demyelination (Minj et al., 2021a,b).

Preliminary research has found and shown the involvement of various molecular signaling targets, as well as their preventive measures, in MND and associated motor neuron disorders such as Alzheimer's, Huntington's, and Parkinson's disease. Scientists worldwide have begun to investigate and discover particular molecular targets as a biomarker in CSF and blood serum to diagnose and cure these neurological dysfunctions. These achievements will aid clinicians, individual drug development, and pharmaceutical research organizations identify therapy and diagnostic procedures.

**Figure 1:**
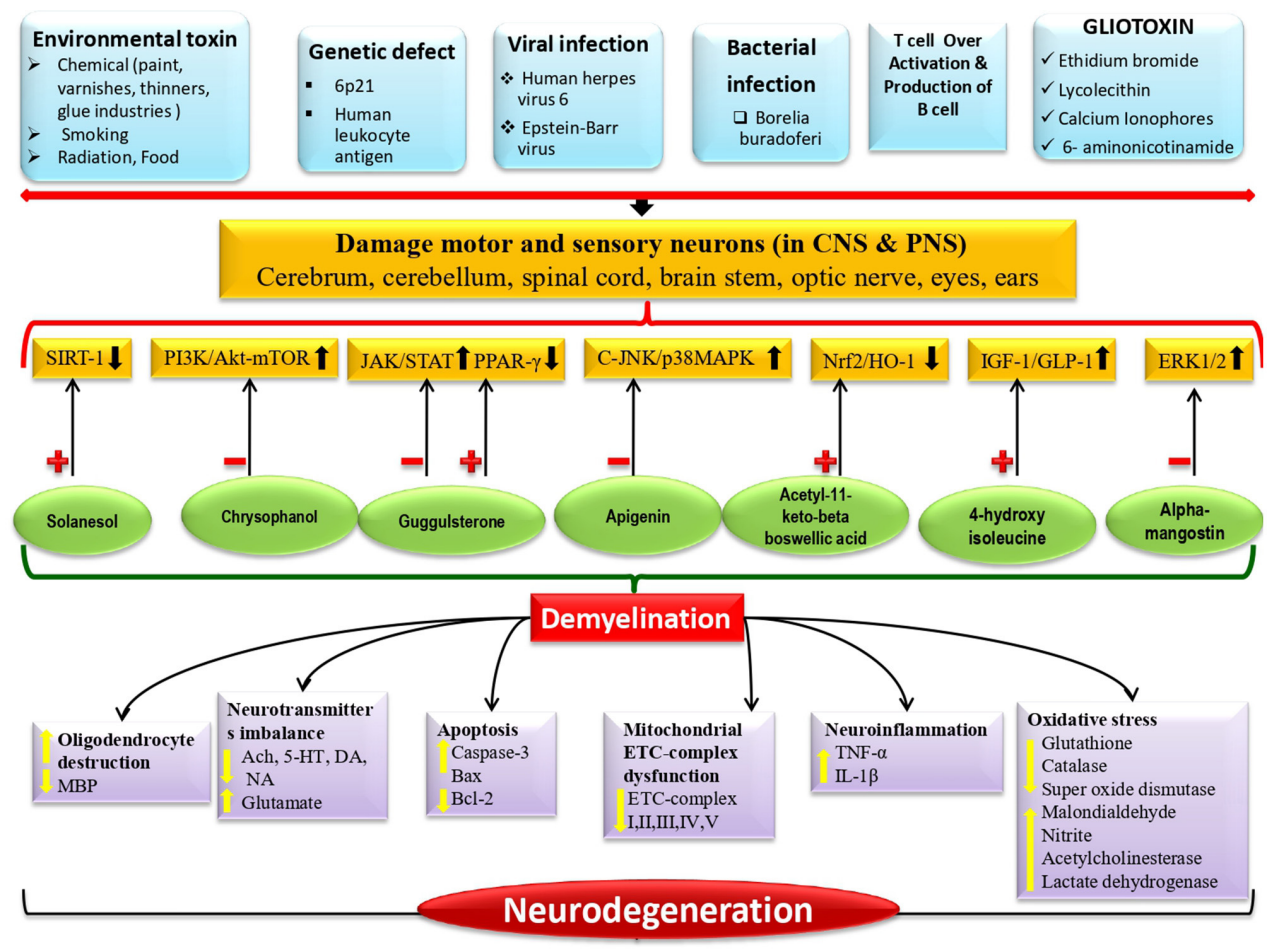
Role of therapeutic modulators by targeting cellular and molecular signaling.

There is clinical and preclinical evidence that cellular and molecular signaling deregulation plays a role in the etiology of MND and similar neuromuscular degenerations. Based on laboratory study findings, researchers chose the following molecular targets as biomarkers and potential signaling modulators to prevent and diagnose MNDs. These cellular and molecular targets, as well as their modulators, can be investigated further in an MND and neuromuscular degenerative condition experimental paradigm as follows: SIRT-1 signaling activator solanesol; Nrf2/HO-1 signaling activator acetyl-11-keto-beta boswellic acid; IGF-1/GLP-1 signaling activator 4-hydroxyisoleucine; ERK1/2 signaling inhibitor alpha-mangostin; JAK-STAT signaling inhibitor and PPAR-⋎ signaling activator guggulsterone; PI3K/Akt/-mTOR signaling inhibitor chrysophanol; and c-JNK/p38MAPK signaling inhibitor Apigenin (Singh et al., 2021; Shandilya et al., 2022; Upadhayay et al., 2022; Yadav et al., 2022). Role of therapeutic modulators by targeting cellular and molecular signaling (Figure 1).

This special issue aims to demonstrate the molecular biomarkers in CSF and blood serum as diagnostic tool. Additionally, to examine the neuroprotective potential of signaling modulators and to understand disease pathways experimentally by performing neurobehavioural, molecular, neurochemical, immunohistopathological, western blot analysis, and gross pathology abnormalities. This special issue also explores the efficacy of signaling modulators in conjunction with currently utilized MND medications and focuses solely on diagnosing motor neuron demyelination and identifying potential treatments to alleviate the neurocomplications associated with MND-like diseases.

## Author contributions

The author confirms being the sole contributor of this work and has approved it for publication.
